# Acculturation of Pacific mothers in New Zealand over time: findings from the Pacific Islands Families study

**DOI:** 10.1186/1471-2458-11-307

**Published:** 2011-05-12

**Authors:** Philip J Schluter, El-Shadan Tautolo, Janis Paterson

**Affiliations:** 1 University of Otago, Department of Public Health and General Practice, Christchurch, New Zealand; 2 AUT University, School of Public Health and Psychosocial Studies, Auckland, New Zealand; 3 The University of Queensland, School of Nursing and Midwifery, Herston QLD 4029, Australia

## Abstract

**Background:**

The epidemiological investigation of acculturation has often been hampered by inconsistent definitions and measurement, and methodological short-comings. Adopting a bi-directional model, with good theoretical and psychometric properties, this study aimed to describe the temporal, ethnic and socio-demographic influences of acculturation for a group of Pacific mothers residing in New Zealand.

**Methods:**

Pacific mothers of a cohort of Pacific infants born at a large tertiary hospital in South Auckland in 2000 were interviewed at 6-weeks, 4-years and 6-years postpartum. At each measurement wave a home interview lasting approximately 90 minutes was conducted with each mother. Adapting the General Ethnicity Questionnaire, two scales of acculturation were elicited: one measuring New Zealand cultural orientation (NZAccult) and one measuring Pacific Islands cultural orientation (PIAccult). Acculturation scores were standardised and analysed using random intercept polynomial and piecewise mixed-effects regression models, accounting for the longitudinal nature of the repeated measured data. Mothers who immigrated to New Zealand and those who lived their lives in New Zealand were investigated separately.

**Results:**

Overall, 1276 Pacific mothers provided 3104 NZAccult and 3107 PIAccult responses over the three measurement waves. Important and significant differences were observed in both bi-directional acculturation measures between the two maternal groups studied. New Zealand cultural orientation increased, on average, linearly with years lived in New Zealand both for immigrant mothers (0.013 per year, 95% CI: 0.012, 0.014), after adjusting for maternal age, and for mothers who lived their lives in New Zealand (0.008 per year, 95% CI: 0.06, 0.010). Immigrant mothers maintained their Pacific cultural orientation for, on average, 12 years before it began to linearly decrease with each year lived in New Zealand thereafter (-0.009 per year, 95% CI: -0.010, -0.008), after adjusting for maternal age. Mothers who lived their lives in New Zealand had a Pacific orientation that was, on average, unchanged regardless of the number of years lived in New Zealand. Significant ethnic and socio-demographic variations were noted.

**Conclusions:**

Understanding the patterns and trajectories of acculturation over time, and its key determinants, is necessary for the development of appropriate targeted health policy and care in typically vulnerable and marginalised immigrant populations.

## Background

Culture and health are intrinsically linked [1-3], with culture acknowledged as an important determinant of health status [3-5]. Also recognised is the importance of migration on health, evolving from seminal New Zealand/Pacific migration studies [6], and early international studies [7,8]. More recent studies have attempted to explain these interrelationships [2,9]. The well-being of a migrant group is determined by interlinking factors that relate to the society of origin, the migration itself, and the society of resettlement [10]. Closely related to culture and migration is the concept of acculturation. Although multiple definitions exist, acculturation has been defined as "culture change that is initiated by the conjunction of two or more autonomous culture systems" [11].

Many studies have investigated and related acculturation to health for minority immigrant groups in areas such as smoking [12], alcohol consumption [13], sexual and reproductive health [14], and nutrition [15]. In most studies, people who have maintained many attitudes and behaviours from their own culture of origin but who have also adopted behaviours and attitudes from the new culture have the best health outcomes. Exceptions have been noted in Pacific mothers and infants in New Zealand [10], Turkish immigrants in both Germany and Canada [16,17], and Hispanic immigrant women in the United States of America (USA) [18]. In these studies, immigrants who retained their own cultural attitudes and behaviours without necessarily adopting attitudes or behaviours from the new culture were more likely to yield more positive health benefits [10,16-18].

However, the epidemiological investigation of acculturation has been hampered by the considerable variation in the definition and measurement of this construct [9]. The literature is replete with models of acculturation, most being multidimensional involving numerous topics and factors [19], but many of the diverse scales employed provide little theoretical orientation, comparability, and have questionable psychometric properties [9,20]. One appealing conceptualisation of acculturation is the bi-directional model proposed by Berry and colleagues [21]. The most influential version of this model is based on the observation that ethnic minorities residing in multicultural societies should confront two essential questions: whether they maintain ethnic identities and whether they want to be actively involved in mainstream culture [22,23]. Attitudes and behaviours towards these two questions conjointly is used to determine cultural orientation; generally classified into four acculturation groups: integration, assimilation, separation and marginalisation [16]. Integration represents people who have maintained many attitudes and behaviours from their culture of origin, but who have also adopted attitudes and behaviours and from the new culture. Assimilation refers to those who have adopted the attitudes and behaviours of the new culture without maintaining the attitudes and behaviours of their culture of origin. Separation occurs when a person fails to recognise or engage with attitudes and behaviours of the new culture and relies on maintaining their own culture of origin. Marginalisation occurs when a person fails to recognise or engage with attitudes and behaviours of the new culture but also fails to maintain attitudes and behaviours from their culture of origin. Berry asserts that acculturative adaptations lead to culture changes in either or both of the migrating and host society groups [16]. Further, it is not inevitable that intergroup contact proceeds uniformly through sequential order to ultimate assimilation; there are various and sometimes unanticipated courses to acculturation which may be bi-directional and reciprocal [16]. Such insights generated by this bi-directional model challenges the ethnic melting-pot assumptions and promotes exploration and resolution of political sensitivities among ethnicities [24]. The epidemiological investigation of acculturation has also been hampered by serious methodological limitations found in previous studies [12,13]. There is a need for longitudinal studies that follow multiple generations of immigrants to understand the temporal influences of acculturation on norms, beliefs and behaviours [13-15].

Within the New Zealand context, Pacific people in New Zealand numbered 266 000 and comprised 6.9% of the population at the 2006 Census [25]. Samoans constituted the largest ethnic group (49.2%), followed by Cook Island Maori (21.8%), and Tongans (19.0%); 60.0% were born in New Zealand; and 65.8% lived in the Auckland urban area [25]. This ethnic diversity is manifest in differing cultures, languages, generations of immigrants, and strength of acculturation [10]. However, Pacific people suffer from an excess of social, health and economic deprivation [26]. There is a growing recognition that issues which have a significant impact on Pacific people's lives need to be understood, of which acculturation stands out [10].

Acculturation is a complex and important concept, yet its epidemiological utility as currently defined and measured remains unclear [9]. As part of a large Pacific birth cohort [27,28], measured over multiple waves, capturing various generations of Pacific immigrant women, and adopting a bi-directional model of acculturation with good theoretical orientation and psychometric properties, this study aimed to describe the temporal, ethnic and socio-demographic influences of acculturation for a group of Pacific mothers residing in New Zealand. This will provide, for the first time, empirical longitudinal data on the pattern and rate of change in acculturation over time for this population; and the identification of important factors affecting these patterns of acculturation.

## Methods

### Study design

The Pacific Islands Families (PIF) study follows a cohort of Pacific infants born at Middlemore Hospital, a large tertiary hospital in South Auckland, between 15 March and 17 December 2000.

### Participants

All potential participants for the PIF study were selected from births where at least one parent was identified as being of Pacific Islands ethnicity and a New Zealand permanent resident. Recruitment occurred through the Birthing Unit and consent was sought to make a home visit. The Birth Unit is used for assessment, induction, birthing or other procedures. For this study, only mothers who self-identified their ethnicity as being Pacific were eligible. As the PIF study follows the child, rather than the mother, only biological, adoptive or step mothers were included within this study.

### Procedure

Approximately 6-weeks after the infant's birth, a female interviewer of matched Pacific Islands ethnicity, fluent in English and the matched Pacific Islands language, visited each mother in her home. Once eligibility was confirmed and written informed consent obtained, the mother participated in an interview lasting approximately 90 minutes concerning her health, family functioning, and the health and development of her child. This interview was conducted in the preferred language of the mother. With written consent, home visits were repeated approximately 4-years and 6-years postpartum. Detailed information about the cohort and methods is described elsewhere [27,28]. Both these articles are available electronically, free of charge, at: http://www.nzma.org.nz/journal/119-1228/1814/content.pdf and http://ije.oxfordjournals.org/content/37/2/273.full.pdf.

### Measures

At each measurement wave the PIF study elicited a suite of questions and, where possible, standardised instruments that were considered relevant and appropriate by both researchers and the Pacific community. These questions, instruments and, where appropriate, response options were included within a booklet. Some questions required additional material, such as flash card with response options, which accompanied this booklet. The interviewers progressed sequentially through the booklet, following clearly defined protocols, reading the questions to the participants and recording their responses. Items elicited included questions on household structure, education and employment, ethnic and cultural identification, length of residency in New Zealand, language use and fluency, parental health and mental health, partner relationships, family finances, housing, transport, church and leisure activities, and various child health and development factors. In all, information on 941 variables of interest was gathered in the 6-weeks home interview. The subsequent home interviews were of similar size. A comprehensive account of these measures appear in the freely available electronic article described above [27].

#### Acculturation measure

Acculturation was conceived and measured using Berry and colleagues' bi-directional model framework [16,21-23,29]. The acculturation instrument chosen for the PIF study was an adaptation of the General Ethnicity Questionnaire (GEQ) [30]. To suit the specific purposes of the PIF study, the scale was shortened and modified thereby developing the Pacific (PIAccult) and New Zealand (NZAccult) versions of the GEQ [10]. The instrument, which appears in the Appendix, was modified to make it relevant to Pacific peoples and New Zealand society as a whole. In shortening the scale, the over-riding objective was to reduce participant burden in an already long questionnaire, without compromising the ability to collect information relevant to the acculturation process and its inter-relationship with the other variables of interest. A detailed account of the measure, its rationale, and an explanation for its modification appears elsewhere [10]. Examination of the adapted acculturation measure yielded positive feedback from both pre-participant focus groups and the Pacific advisory group; an independent group of community representatives who provide guidance on the scientific and cultural directions of the research [10]. The internal consistency of the measure was also examined, using Cronbach's α, and was found to be acceptable (α = 0.81 and 0.83 for the NZAccult and the PIAccult scales, respectively) [10]. For the purpose of this study, the standardised NZAccult and PIAccult scores were analysed, rather than the four acculturation classifications which are generally used.

#### Selected socio-demographics variables

Acculturation has been shown to be influenced by a number of socio-demographic and life-style factors. Here, self-reported ethnicity, marital status, highest educational qualification, smoking status, employment status, usual number of household residents and household income were investigated. As these variables can vary over time, responses elicited at each measurement wave were used in the analyses (except for ethnicity and household income where baseline 6-weeks values were used). Acculturation has been associated with harmful smoking behaviours in Asian women in the USA [12], and increased smoking among Hispanic women in the USA [31]. Socio-economic position, such as educational qualification, employment status, and income have been associated with acculturation status - often in a complex fashion [32]. Marital status and usual household size were *a priori *hypothesised to be associated with acculturation, as these significant people (where present) may support maintenance or change in participants' cultural values and practices. Ethnicity was also studied as there are considerable differences between Pacific cultures and characteristics [10], yet a paucity of empirical information. Moreover the failure to disaggregate the various Pacific sub-ethnicities not only disguises the heterogeneity of the Pacific population in New Zealand but is offensive to some Pacific people who value the uniqueness of their cultures and languages as much as other ethnic groups [33].

### Statistical analysis

All biological, adoptive and step mothers of Pacific ethnicity in the PIF study were identified and included in the analyses. Mothers were classified into one of two groups: (i) those who lived their lives in New Zealand (defined as having spent within 2 years of their maternal age in New Zealand); and (ii) those who immigrated to New Zealand. Standardised NZAccult and PIAccult scores for each participant over each measurement wave was determined by summing the Likert response scores (range: 1-5) over the 11 questions and then transforming these sums to the lie on the unit (0-1) interval. Spearman's correlation was used to estimate the crude associations between these standardised scores. Individual line graphs of the standardised scores over years lived in New Zealand were then plotted for a sample of mothers, together with superimposed lowess curves (non-parametric mean estimator functions) of all mothers to guide the pattern and polynomial order of pursuant regression models.

Recognising the longitudinal nature of participant's self-reported NZAccult and PIAccult scores, and that the length of exposure to culture within New Zealand was different for each study participant, random intercept mixed-effects regression models were employed. Initially, models were developed to explore the relationship between years Pacific mothers spent in New Zealand and the standardised acculturation scores for the two maternal classification groups (i.e. those who immigrated to New Zealand and those who lived their lives in New Zealand). Maternal age, centred on its baseline mean value, was also included as a covariate in the analyses of those who immigrated to New Zealand. However, as maternal age and years in New Zealand was, by definition, exchangeable for Pacific mothers who lived their lives in New Zealand, maternal age was not included in these analyses. The variation between mothers was modelled by the random intercept. As the lowess curve for the standardised PIAccult scores of Pacific mothers who immigrated to New Zealand appeared to have a two-piece linear function over time [34], this was assessed against polynomial regressions of various orders using the Bayesian Information Criterion (BIC) statistics. The BIC can be used to determine model superiority between nested, non-nested and partially-nested competing parametric regression models with different numbers of parameters or variable forms [35]. The BIC penalises for model complexity and rewards for goodness-of-fit; with the preferred model balancing these competing demands and yielding the lowest value. Once the mixed-effects models were determined, standardised residuals were calculated and checks undertaken (histograms and plots of observed values against expected values) to ascertain whether any important violations of the model assumptions could be seen. Expected values over years spent in New Zealand from these mixed-effects models calculated at the centred maternal age value were then added to the line and lowess plots described above. The effect of selected socio-demographic variables on the standardised acculturation score, after adjusting for years spent in New Zealand (and maternal age in the immigrant mother models), were then separately investigated in each model. Wald's χ^2 ^test was used to assess the significance of these variables. All analyses and graphics were conducted using Stata version 10.0 (StataCorp, College Station, TX, USA), and α = 0.05 defined statistical significance.

### Ethics

Ethical approval was obtained from the Auckland Branch of the National Ethics Committee, the Royal New Zealand Plunket Society and the South Auckland Health Clinical Board. Conduct of the study complied with the ethical standards for human experimentation as established by the Helsinki Declaration.

## Results

In total, 1477 mothers were eligible for the PIF study, 1376 (93.2%) participated at the baseline 6-weeks interview, 1048 (71.0%) completed the 4-years interview, and 1001 (67.8%) completed the 6-years interview [27,28]. However, at the baseline 6-weeks interview, 99 (7.2%) mothers self-identified their ethnicity as being non-Pacific, eligible for the PIF study due to the Pacific identity of the child's father, but ineligible for this study. Moreover, 1 (0.1%) participant reported as not being a biological, adoptive or step mother, and so was excluded. This left 1276 mothers at the baseline 6-weeks interview, 951 at the 4-years interview, and 894 at the 6-years interview eligible for this study. Of these participants, 1269 (99.5%) were birth mothers, 359 (28.1%) were New Zealand born, and the average age at the baseline 6-weeks interview was 28.5 years. The eligible participants' baseline socio-demographic distributions appear in Table 1, together with the socio-demographic breakdowns by the two maternal groups.

**Table 1:** Socio-demographics of participating Pacific mothers at the 6-weeks measurement waves (n = 1276) partitioned by those who immigrated to New Zealand and for those who have lived their lives entirely within New Zealand

	Total		Immigrated to New Zealand		Lived entirely within New Zealand	
	n	(%)	n	(%)	n	(%)
*Age (years)*						
<20	93	(7.3)	36	(4.0)	57	(15.0)
20-24	324	(25.4)	174	(19.4)	150	(39.4)
25-29	342	(26.8)	229	(25.6)	113	(29.7)
30-34	312	(24.5)	269	(30.1)	43	(11.3)
35-39	163	(12.8)	147	(16.4)	16	(4.2)
≥40	42	(3.3)	40	(4.5)	2	(0.5)
*Ethnicity*						
Samoan	649	(50.9)	467	(52.2)	182	(47.8)
Tongan	289	(22.6)	258	(28.8)	31	(8.1)
Cook Island Maori	232	(18.2)	116	(13.0)	116	(30.4)
Other Pacific	106	(8.3)	54	(6.0)	52	(13.6)
*Marital status*						
Married/de facto	1027	(80.5)	746	(83.4)	281	(73.8)
Single	249	(19.5)	149	(16.6)	100	(26.2)
*Highest educational qualifications*						
No formal qual.	506	(39.7)	395	(44.1)	111	(29.1)
Secondary	439	(34.4)	308	(34.4)	131	(34.4)
Post-secondary	331	(25.9)	192	(21.5)	139	(36.5)
*Smoking status*						
Non-smoker	968	(76.0)	743	(83.3)	225	(59.1)
Smoker	305	(24.0)	149	(16.7)	156	(40.9)
*Employment status*						
Full-time employed	52	(4.1)	38	(4.2)	14	(3.7)
Part-time employed	24	(1.9)	18	(2.0)	6	(1.6)
Not employed	1200	(94.0)	839	(93.7)	361	(94.8)
*Number of usual household residents (including mother and child)*						
2-4	250	(19.6)	176	(19.7)	74	(19.4)
5-7	646	(50.7)	455	(50.9)	191	(50.1)
≥8	379	(29.7)	263	(29.4)	116	(30.4)
*Household income (NZD)*						
≤$20 000	427	(33.5)	282	(31.5)	145	(38.1)
$20 001-$40 000	660	(51.7)	484	(54.1)	176	(46.2)
>$40 000	146	(11.4)	102	(11.4)	44	(11.6)
Unknown	43	(3.4)	27	(3.0)	16	(4.2)

*Within 2 years of maternal age.

Overall, the ethnic frequencies in Table 1 were broadly similar to those seen in the general New Zealand Pacific population [23,24]. The sample include 895 (70.1%) Pacific mothers who had immigrated to New Zealand and 381 (29.9%) mother who had spent their life in New Zealand. For the mothers who immigrated, their median length of time in New Zealand at the baseline 6-weeks interview was 11 years (Q_1 _= 5 years, Q_3 _= 15 years).

Clear differences in the socio-demographic profiles were observed between the two maternal groups in Table 1. Mothers who immigrated were generally older, more likely to be of Tongan ethnicity and less likely to be of Cook Island Maori ethnicity, more likely to be partnered, more likely to have no formal qualifications, and less likely to smoke than their New Zealand living counterparts.

### Standardised NZAccult and PIAccult scores

Overall, 1276 Pacific mothers provided 3104 valid NZAccult responses (average 2.4 per mother) and 3107 valid PIAccult responses (average 2.4 per mother) over the three measurement waves. For Pacific mothers who immigrated to New Zealand, the median standardised NZAccult and PIAccult score over all three measurement waves was 0.48 (Q_1 _= 0.36, Q_3 _= 0.64) and 0.82 (Q_1 _= 0.73, Q_3 _= 0.89), respectively. For Pacific mothers who had lived their lives in New Zealand, the median standardised NZAccult and PIAccult score over all three measurement waves was 0.70 (Q_1 _= 0.61, Q_3 _= 0.80) and 0.61 (Q_1 _= 0.41, Q_3 _= 0.77), respectively. Spearman's correlation between standardised NZAccult and PIAccult scores for Pacific mothers who immigrated to New Zealand was -0.22 and for Pacific mothers who had lived their lives in New Zealand was 0.10.

### Standardised NZAccult scores over time

**Figure 1:**
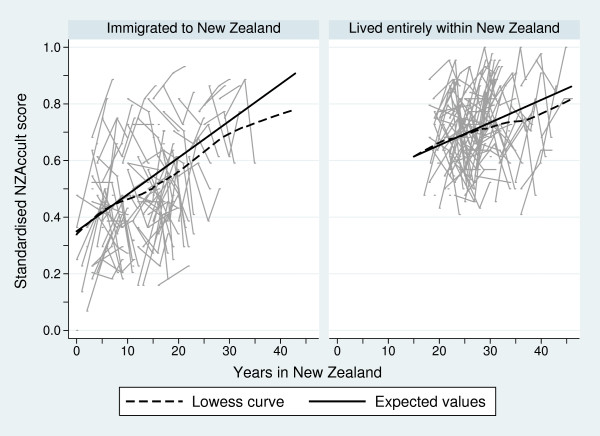
Individual line graphs of the standardised NZAccult score over years lived in New Zealand for a sample of 100 mothers for who immigrated to New Zealand and for a sample of 100 mothers who have lived their entire lives in New Zealand, together with superimposed lowess curves (dashed lines) and the expected values (solid lines) at maternal age 28.5 years from the mixed-effects models for the full samples

Figure 1 presents individual line graphs of the standardised NZAccult scores over years lived in New Zealand for the two maternal groups, together with superimposed lowess curves. While there is considerable variability in standardised NZAccult score responses over time, the lowess curves depict an approximately linear increase in scores with years lived in New Zealand for both groups of participants.

The mixed-effects regression model, treating mothers as random-effects, confirmed these linear relationships. Table 2 houses the regression estimates for the fixed-effects components. Mothers aged 28.5 years at the baseline 6-weeks measurement wave who immigrated to New Zealand had an average increase in NZAccult scores of 0.013 for every year they resided in New Zealand. However, this linear increase was mitigated by maternal age - with older mothers having, on average, a reduced increase in NZAccult scores and younger mothers having, on average, a larger increase in scores. Mothers who lived their lives entirely within New Zealand had an average increase in NZAccult scores of 0.008 for every year they resided in New Zealand. The expected values over years in New Zealand from these mixed-effects models have also been superimposed on Figure 1. The estimated standard deviation for the random-effects between mothers who immigrated to New Zealand was 0.094 (95% confidence interval [CI]: 0.086, 0.103) and between mothers who lived their lives in New Zealand was 0.117 (95% CI: 0.111, 0.125). This implies that Pacific mothers who lived their lives within New Zealand had a more variable response pattern to the NZAccult questions than those who immigrated.

**Table 2:** Regression coefficients and associated 95% confidence intervals (95% CI) of mixed-effects models relating time in New Zealand to standardised NZAccult and PIAccult scores for Pacific mothers who immigrated to New Zealand and for those who have lived their lives entirely within New Zealand

	Immigrated to New Zealand*		Lived entirely within New Zealand	
	Est.	(95% CI)	Est.	(95% CI)
*Standardised NZAccult score*				
Intercept	0.350	(0.334, 0.366)	0.494	(0.439, 0.550)
Time in New Zealand (years)	0.013	(0.012, 0.014)	0.008	(0.006, 0.010)
Maternal age (years) - centred at 28.5 years	-0.007	(-0.008, -0.006)		
*Standardised PIAccult score*				
Intercept	0.809	(0.801, 0.818)	0.626	(0.611, 0.641)
Time in New Zealand after 12-years (years)	-0.009	(-0.010, -0.008)		
Maternal age (years) - centred at 28.5 years	0.005	(0.004, 0.006)		

*adjusted for maternal age.

### Standardised PIAccult scores over time

**Figure 2:**
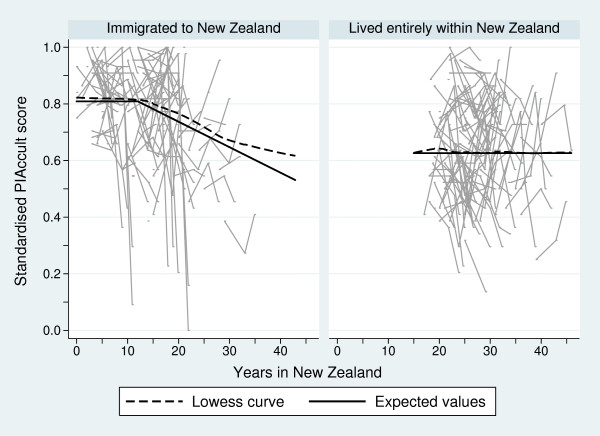
Individual line graphs of the standardised PIAccult score over years lived in New Zealand for a sample of 100 mothers for who immigrated to New Zealand and for a sample of 100 mothers who have lived their entire lives in New Zealand, together with superimposed lowess curves (dashed lines) and the expected values (solid lines) at maternal age 28.5 years from the mixed-effects models for the full samples

Figure 2 presents individual line graphs of the standardised PIAccult score over years lived in New Zealand for the two maternal groups, together with superimposed lowess curves. Like the standardised NZAccult scores, there is considerable variability in the individual response profiles over time. However, unlike the standardised NZAccult scores, the lowess curves depict different patterns between groups. For those Pacific mothers who immigrated to New Zealand, a piecewise trend over years in New Zealand was seen. Specifically, mothers living 12 years or less in New Zealand had a relatively constant average standardised PIAccult score, which then decreased in an approximately linear fashion for every additional year lived in New Zealand thereafter. However, for Pacific mothers who have lived their lives in New Zealand there appeared to be an unchanging average standardised PIAccult score over all years they lived in that nation.

These relationships were confirmed in the ensuing mixed-effects regression analyses; with regression estimates of the fixed-effects components also included in Table 2. Mothers aged 28.5 years at the baseline 6-weeks measurement wave who immigrated to New Zealand had, on average, no change in PIAccult scores for the first 12 years they resided in New Zealand but then a decrease in PIAccult scores of 0.009 for every additional year they resided in New Zealand thereafter. Again this effect was mitigated by maternal age - with older mothers having, on average, a reduced decrease in PIAccult scores and younger mothers having, on average, a larger decrease in scores. This piecewise model yielded a BIC statistic 29.5 lower than the second order polynomial mixed-effects regression model, and 53.1 lower than the third order polynomial mixed-effects regression model; and was thus preferred. The choice of the 12-years threshold was based on visual inspection of the lowess curve in Figure 2, and noting that the 11-years threshold piecewise model yielded a BIC statistic 1.4 higher and the 13-years threshold model yielded a BIC statistic approximately equivalent to the 12-years threshold model but produced expected values that appeared to over-shoot the change-point seen in the lowess curve (Figure 2). Mothers who lived their lives entirely within New Zealand had, on average, no change in PIAccult scores regardless of the number of years they resided in New Zealand. To aid interpretation, the expected values over years in New Zealand from these mixed-effects models have been superimposed on Figure 2. The estimated standard deviation for the random effects between mothers who immigrated to New Zealand was 0.074 (95% CI: 0.067, 0.081) and between mothers who lived their lives in New Zealand was 0.129 (95% CI: 0.117, 0.143). Again, this implies that Pacific mothers who lived their lives within New Zealand had a more variable response pattern to the PIAccult questions than those who immigrated.

### Associations with NZAccult and PIAccult scores

Table 3 reports the regression coefficients and associated standard errors for a selection of socio-demographic factors individually added to the mixed-effects models relating time in New Zealand to standardised NZAccult and PIAccult scores presented in Table 2. Only marital status was not significantly related to NZAccult or PIAccult scores for both maternal groups. Generally, Samoan and Tongan mothers had significantly higher PIAccult scores than other Pacific ethnic groups; mothers with lower educational qualifications had lower NZAccult scores than mothers with higher educational qualifications; employed mothers had higher NZAccult scores than unemployed mothers - but no difference in PIAccult scores; mothers in larger households had lower NZAccult scores and higher PIAccult scores than mothers in smaller households; and immigrant mothers with lower household income had lower NZAccult scores and higher PIAccult scores than those in the highest income bracket. However, household income had no significant association with either NZAccult or PIAccult scores for mothers who lived their lives in New Zealand.

**Table 3:** Regression coefficients and associated standard errors (S.E.) for a selection of socio-demographic factors individually added to the mixed-effects models relating time in New Zealand to standardised NZAccult and PIAccult scores presented in Table 2

	Standardised NZAc cult score						Standardised PIAcc ult score					
	Immigrated to New Zealand			Lived entirely within New Zealand			Immigrated to New Zealand			Lived entirely within New Zealand		
	Est.	(S.E.)	P-value	Est.	(S.E.)	P-value	Est.	(S.E.)	P-value	Est.	(S.E.)	P-value
*Ethnicity*			<0.001			0.12			<0.001			<0.001
Samoan	(reference)			(reference)			(reference)			(reference)		
Tongan	-0.033	(0.010)		-0.005	(0.021)		-0.050	(0.007)		-0.038	(0.027)	
Cook Island Maori	0.039	(0.013)		-0.027	(0.012)		-0.132	(0.010)		-0.129	(0.016)	
Other Pacific	0.076	(0.019)		0.004	(0.016)		-0.107	(0.014)		-0.129	(0.022)	
*Marital status*			0.69			0.46			0.19			0.07
Married/de facto	(reference)			(reference)			(reference)			(reference)		
Single	-0.004	(0.010)		-0.008	(0.011)		-0.011	(0.008)		-0.022	(0.012)	
*Highest educational qualifications*			<0.001			0.001			0.004			0.31
No formal qual.	-0.123	(0.009)		-0.045	(0.012)		0.026	(0.008)		-0.024	(0.016)	
Secondary	-0.059	(0.010)		-0.009	(0.011)		0.012	(0.008)		-0.006	(0.014)	
Post-secondary	(reference)			(reference)			(reference)			(reference)		
*Smoking status*			0.07			0.74			0.008			0.06
Non-smoker	(reference)			(reference)			(reference)			(reference)		
Smoker	0.018	(0.010)		-0.003	(0.010)		-0.021	(0.008)		-0.022	(0.012)	
*Employment status*			<0.001			<0.001			0.28			0.80
Full-time employed	0.078	(0.009)		0.053	(0.011)		0.009	(0.007)		0.006	(0.011)	
Part-time employed	0.054	(0.013)		0.044	(0.016)		-0.009	(0.011)		-0.005	(0.016)	
Not employed	(reference)			(reference)			(reference)			(reference)		
*Number of usual household residents*			<0.001			0.21			0.003			0.02
2-4	(reference)			(reference)			(reference)			(reference)		
5-7	-0.018	(0.010)		-0.015	(0.011)		0.027	(0.008)		0.031	(0.012)	
≥8	-0.047	(0.011)		-0.022	(0.013)		0.023	(0.009)		0.033	(0.014)	
*Household income (NZD) at baseline*			0.02			0.63			0.009			0.25
≤$20 000	-0.049	(0.015)		0.011	(0.018)		0.009	(0.012)		-0.009	(0.026)	
$20 001-$40 000	-0.034	(0.014)		0.016	(0.017)		0.021	(0.011)		0.000	(0.025)	
>$40 000	(reference)			(reference)			(reference)			(reference)		
Unknown	-0.026	(0.028)		0.037	(0.030)		-0.037	(0.022)		-0.079	(0.044)	

## Discussion

Important and significant differences were observed in both bi-directional acculturation measures between the two maternal immigration groups studied. Length of time in New Zealand was, on average, linearly and positively associated with increased standardised NZAccult scores. The baseline average was lower for mothers who immigrated to New Zealand compared to those who lived their lives entirely within the nation, but their rate of increase was greater. Pacific mothers' length of exposure to New Zealand's peoples and culture appeared to proportionally affect their orientation towards these peoples and culture. Even more notable were the standardised PIAccult score relationships identified. The piecewise relationship identified for mothers who immigrated to New Zealand suggests that Pacific mothers generally maintain their strong Pacific cultural alignment for approximately 12 years, after which time the strength of this alignment linearly decreases and approaches the level observed in mothers who have lived their lives entirely within New Zealand. Furthermore, the average standardised PIAccult score was static for Pacific mothers who have lived their lives entirely within New Zealand, regardless of their age. Understanding these time-dependent relationships is vital if efficacious ethnically or culturally targeted health promotion policies and strategies are to be developed and promulgated nationally [36] or internationally [37].

The pattern of acculturation identified for the immigrant mother group is consistent with some cross-sectional findings demonstrated elsewhere. For example, in the international study of immigrant youth [38], there was increase in acculturation to the 'national' way with exposure (from 6% to 15% to 24%) of the sample, but much less decrease in the 'ethnic' way (from 26% to 23% to 20%). These findings demonstrate the importance and necessity of assessing the two dimensions of acculturation separately.

Important and significant ethnic and socio-demographic differences were also uncovered, particularly for the immigrant Samoan and Tongan mothers. Previous research has indicated that the many first-generation Samoan and Tongan migrants have not embraced *palangi *(European) culture to the detriment of their own [10]. Many first-generation migrants to New Zealand retain close links with their family in practical ways, such as returning to visit relatives in Tonga and sending remittances [39]. For these migrants their cultural identity is continually reinforced through their participation in institutions, such as church and adherence to certain cultural practices and maintenance of their Pacific language at home [40,41]. However, the strength of connection to culture and language by some of the New Zealand-born migrants in the subsequent generations is weakening, as they struggle with maintaining a sense of Pacific identity within the New Zealand society [40]. However, much more population-based health research among Samoans and other Pacific groups has been advocated and is required to fully understand these relationships and their implications [41].

The interactions between migration and health is extremely complex, context specific, and at times appears counter-intuitive [32,42]. However, the health impact of migration and acculturation is heavily conditioned by socio-demographic factors [32,42]. With respect to the selected socio-demographic variables investigated within this study, the smoking status of Pacific mothers was significantly associated with acculturation - consistent with previous research in women elsewhere [12,27]. However, the relationship was not uniform across both cultural orientation groups. Currently smoking Pacific mothers had, on average, a significantly lower Pacific cultural alignment score than their non-smoking counterparts but there was no difference between their New Zealand cultural orientation scores. This supports the supposition that it is the retention of strong cultural attitudes and behaviours that is more likely to yield more positive benefits rather than the adoption of behaviours and attitudes from the new culture [10,16-18]. Again, these findings demonstrate the importance of assessing the two dimensions of acculturation.

Generally, mothers who immigrated to New Zealand in a lower socio-economic position (measured by highest educational qualification, employment status, and household income) had significantly lower New Zealand and higher Pacific acculturation scores than those in higher positions. However, apart from highest educational qualification and employment status in the New Zealand scores, these patterns had largely disappeared for mothers who lived their lives in New Zealand. Many studies indicate that the health of its population is strongly tied to their economic conditions and inequalities [37]. If the retention of immigrants' attitudes and behaviours is protective, then this presents a potential avenue for culturally appropriate family or community level prevention and wellness efforts, especially amongst those in the more disadvantaged economic positions [32].

Longitudinal studies of acculturation are rare and much needed to improve both the empirical public health research base and our population level understanding of its implications on health. This large, scientifically robust longitudinal study squarely addresses identified serious deficiencies in many acculturation studies found within public health research [9,12-14]. A bi-directional acculturation measure was employed that has considered theoretical orientation and underpinning [16], with good psychometric properties [10]; and contemporary apposite methods of analysis carefully undertaken, accounting for piecewise relationships observed - a phenomenon that is not uncommon, but rarely modelled [34]. However, the presented study is not without limitations. In particular, we were unable to distinguish between second, third and higher generational Pacific mothers; the presented results are unlikely to be generalisable to different immigrant groups situated in this or other countries; and a longer individual follow-up time would yield further valuable information. Furthermore, by virtue of data availability, the presented analyses were focused on mothers. Additional information that captured acculturation measures of mothers' partners, their families, and their communities would add further insight and likely explain the considerable variation seen in the various standardised NZAccult and PIAccult scores.

Another important limitation is the choice and continued use of the acculturation instrument embedded within the PIF study; a choice made over a decade ago [10]. Common to population longitudinal studies which span many years, appropriate instruments chosen at inception for repeated elicitation are at risk for becoming obsolete in the ensuing years. Conversely, changing instruments between measurement waves typically introduces considerable conceptual and analytical difficulties. As such, more recent and arguably richer acculturation models and instruments that have propounded were not utilised within the PIF study. These include, for example, domain specific models [43,44], specialised acculturation and integration concepts such as cognitive-evaluative, functional specialisation, frame switching and constructive marginalisation models [45], or intersubjective perceptions approaches [46]. In particular, the utilised instrument ignores the psychological component of acculturation (the emotions, values, and other psychological changes that might result from cultural contact) in preference for the sociocultural component (the acquisition of competence in social and cultural practices such as language, food, and lifestyle). Clearly, an improved understanding of acculturation would result if both components were adequately captured and reliably measured.

Understanding the acculturation of immigrant populations is fundamentally important [37], especially with its link to health and well-being of the individual and communities. There is mounting evidence demonstrating that traditional cultural alignment has improved health outcomes [10,16-18]. Also vital and necessary is an understanding of the patterns and trajectories of acculturation over time, and its key determinants, in the development of appropriate health policy and the targeted provision of health promotion and care in typically vulnerable and marginalised populations.

## Conclusions

While immigration and acculturation are intrinsically linked with health, the epidemiologic investigation of acculturation has been hampered by inconsistent definitions and measurement, and often by significant methodological limitations. Using a bi-directional measure with good theoretical orientation, conceptual underpinning, and psychometric properties, acculturation is assessed in this large cohort study of Pacific Island mothers over three measurement waves spanning six years. Significant and ethically differential patterns of acculturation were found and described between immigrant and New Zealand born mothers. Understanding and then tailoring health promotion efforts to recognise and meet the needs of different communities is one important step in reducing health inequalities confronting many immigrant populations.

## List of abbreviations used

NZAccult: New Zealand cultural orientation; PIAccult: Pacific Islands cultural orientation; PIF: Pacific Islands Families; GEQ: General Ethnicity Questionnaire; BIC: Bayesian Information Criterion; USA: United States of America; CI: confidence interval; Est: estimate; S.E.: standard error; N.Z.: New Zealand; NZD: New Zealand dollar.

## Competing interests

The authors declare that they have no competing interests.

## Authors' contributions

All authors (PJS, EST, JP) participated in the planning and conception of the research questions and the study design. PJS was responsible for retrieving and analysing the data. PJS drafted the article, and all authors participated in interpreting the data and critically revising the manuscript for important intellectual content. All authors read and approved the revised manuscript.

## Appendix

### Pacific Islands (PIAccult) and New Zealand (NZAccult) Acculturation Scales

#### The PIAccult (Pacific orientation)

[a] I was brought up the Pasifika way

[b] I am familiar with Pasifika practices and customs

[c] I can understand a Pasifika language well

[d] I have several Pasifika friends

[e] Most of my friends speak a Pasifika language

[f] I participate in Pasifika sports and recreation

[g] I speak a Pasifika language

[h] I have contact with Pasifika families and relatives

[i] I eat Pasifika food

[j] I visit a traditional Pasifika healer when I have an illness

[k] I go to a church that is mostly attended by Pasifika people

#### The NZAccult (New Zealand orientation)

[a] I was brought up the New Zealand way

[b] I am familiar with New Zealand practices and customs

[c] I can understand English well

[d] I have several non Pasifika friends

[e] Most of my friends speak English

[f] I participate in New Zealand sports and recreation

[g] I speak English

[h] I have contact with non-Pasifika families and relatives

[i] I eat non-Pasifika food

[j] I visit western-trained doctors when I have an illness

[k] I go to a church that is mostly attended by non-Pasifika people

The PIAccult instrument was elicited first. The interviewer says "I will read a list of statements. Please tell me how much you agree or disagree with each one using the following scale (1) Strongly disagree (2) Disagree (3) Neither disagree or agree (4) Agree (5) Strongly agree". A card showing these response options is given to the participant and questions [a] to [e] are then read aloud and responses recorded. Next the interview says "Now using this card which has response options (1) Not at all (2) A little (3) Somewhat (4) Quite a lot (5) A lot, please tell me how much or how often you do the following things." A card showing these response options is given to the participant and questions [f] to [k] are then read aloud and responses recorded. The process is then repeated for the NZAccult instrument.

## Pre-publication history

The pre-publication history for this paper can be accessed here:

http://www.biomedcentral.com/1471-2458/11/307/prepub
